# Recent Advances in Multi-Material 3D Printing of Functional Ceramic Devices

**DOI:** 10.3390/polym14214635

**Published:** 2022-10-31

**Authors:** Hui Chen, Liang Guo, Wenbo Zhu, Chunlai Li

**Affiliations:** 1 Changchun Institute of Optics, Fine Mechanics and Physics, Chinese Academy of Sciences, Changchun 130033, China; 2 University of Chinese Academy of Sciences, Beijing 100049, China

**Keywords:** multi-material, 3D printing, functional ceramic devices, capacitors, multilayer substrates, microstrip antennas

## Abstract

In recent years, functional ceramic devices have become smaller, thinner, more refined, and highly integrated, which makes it difficult to realize their rapid prototyping and low-cost manufacturing using traditional processing. As an emerging technology, multi-material 3D printing offers increased complexity and greater freedom in the design of functional ceramic devices because of its unique ability to directly construct arbitrary 3D parts that incorporate multiple material constituents without an intricate process or expensive tools. Here, the latest advances in multi-material 3D printing methods are reviewed, providing a comprehensive study on 3D-printable functional ceramic materials and processes for various functional ceramic devices, including capacitors, multilayer substrates, and microstrip antennas. Furthermore, the key challenges and prospects of multi-material 3D-printed functional ceramic devices are identified, and future directions are discussed.

## 1. Introduction

**Figure 1:**
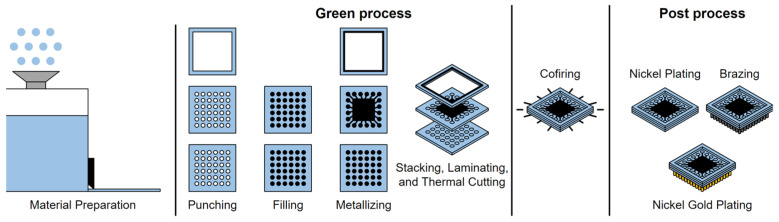
Cofired ceramic process.

Compared with structural ceramics, functional ceramic devices are characterized by their detection, transformation, coupling, transmission, processing, and storage of information (i.e., electrical, magnetic, optical, acoustic, thermal, force, and biological [1]), and they have been used in numerous fields (i.e., aviation, automobile, integrated circuit, communication, medical, and energy) [2]. Most functional ceramic devices, such as multilayer ceramic capacitors, multilayer ceramic substrates, filters, chip antennas, power dividers, and duplexers, can be fabricated by a high-temperature cofired ceramic (HTCC) process or low-temperature cofired ceramic (LTCC) process [3,4,5,6,7,8]. These processes require multiple steps: (1) material preparation for ceramic tapes and functional pastes, (2) punching of the green tapes for via formation, (3) filling and metalizing for vertical conductors and horizon circuitries formation, (4) stacking and laminating for 3D ceramic green body formation that can be maintained as an array or cut into individual products, (5) cofiring, and (6) post processing including nickel plating, brazing, and gold plating (Figure 1) [9]. As a result, the metal pastes (e.g., silver, gold, tungsten, and molybdenum) and resistor pastes can be cofired with the ceramic body to fabricate multilayer ceramic components. With the continuous development of electronic science and technology, the HTCC and LTCC processes are poorly suited to the rapid prototyping and low-cost manufacturing of miniaturized, thin, refined, and highly integrated functional ceramic devices.

Three-dimensional printing is a breakthrough manufacturing method that can build 3D objects layer-by-layer from digital models and has developed rapidly since the 1980s. Three-dimensional printing is commonly known as additive manufacturing (AM), and the ASTM/ISO standard for AM classifies the different technologies into seven subcategories: material extrusion, material jetting, binder jetting, vat photopolymerization, powder bed fusion, direct energy deposition, and sheet object lamination [10]. According to this classification, HTCC and LTCC based on green tape lamination can also be considered an AM process, though different from 3D printing; therefore, to distinguish this concept, we use the term “3D printing” rather than “AM”.

**Figure 2:**
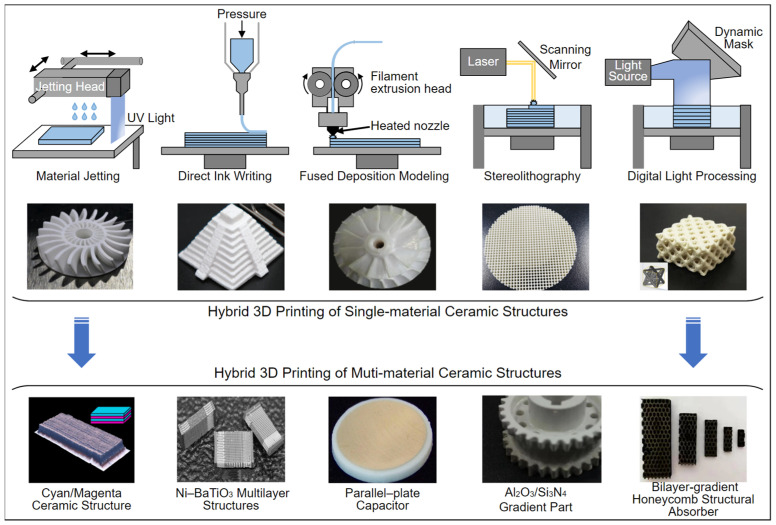
Schematics of five different 3D printing techniques, including material jetting (MJ), direct ink writing (DIW), fused deposition modeling (FDM), stereolithography (SLA), and digital light processing (DLP), and their applications in single material and multi-material ceramic 3D structures. Schematic images are adapted from [12,13,14,15,16,17,18,19,21,22,23].

In recent years, 3D printing has become a research focus in fine ceramic manufacturing, and it can be used to construct most structural ceramics [11,12,13,14]. However, functional ceramic devices with a composite structure comprising two or more materials usually contain strip lines, microstrip lines, and vias; as a result, in addition to enhancing the spatial resolution and printing speed, several problems in terms of the raw materials, printing strategies, and sintering process still need to be solved. Multi-material 3D printing of functional ceramic devices is in early development and has great research motivation and application potential [15,16,17,18,19]. Figure 2 shows several major 3D printing techniques, including material jetting (MJ), direct ink writing (DIW), fused deposition modeling (FDM), stereolithography (SLA), and digital light processing (DLP) [20]. Among them, MJ, DIW, and FDM can independently accomplish a multi-material 3D printing task by simply equipping the same number of nozzles or jetting heads as the material being processed because these techniques deposit materials directly to the desired voxel. However, SLA and DLP generally need to be combined with MJ, DIW, or other special techniques (e.g., sputtering and plating) to integrate multiple materials together into one object [21]. We review the latest progress in multi-material 3D printing for functional ceramic devices, including capacitors, multilayer substrates, and microstrip antennas, based on MJ, DIW, FDM, SLA, DLP, and a hybrid process simultaneously employing multiple techniques, and identify existing problems and future development directions for multi-material 3D printing of functional ceramic devices, which is expected to support future research.

## 2. Multi-Material 3D Printing Methods

### 2.1. Material Jetting (MJ)

MJ derives from conventional 2D ink jetting extensively used in graphic fields (e.g., office documents, digital photos, labels, tiles, and clothing) [24]. In MJ, droplets of the feedstock material are selectively deposited and solidified in successive layers [25]. Several MJ techniques are recognized by the mechanism of droplet generation, in which the most widely used are inkjet printing (IJP), aerosol jet printing (AJP), and electrohydrodynamic inkjet printing (EHDP). Over the past few years, these MJ techniques have attracted interest in the on-demand fabrication of highly customizable electronics because of their abilities to precisely and smartly deliver high-resolution patterns with a diversified structure (<1 µm) from design files in a non-contact manner. Multi-material 3D printing of supercapacitors [26], filters [27], tapered optical waveguides [28], solar cells [29], microsensors [30], flexible circuits [31], and high-density redistribution layers (RDLs) of silicon interposers [32] has been easily implemented by following the same approach of conventional 2D ink jetting by sequential deposition of functional inks via an MJ system equipped with multiple jetting heads. Table 1 lists several recently developed functional inks whose printing methods, applications, sintering or curing techniques, and electrical properties are summarized.

#### 2.1.1. Inkjet Printing (IJP)

**Figure 3:**
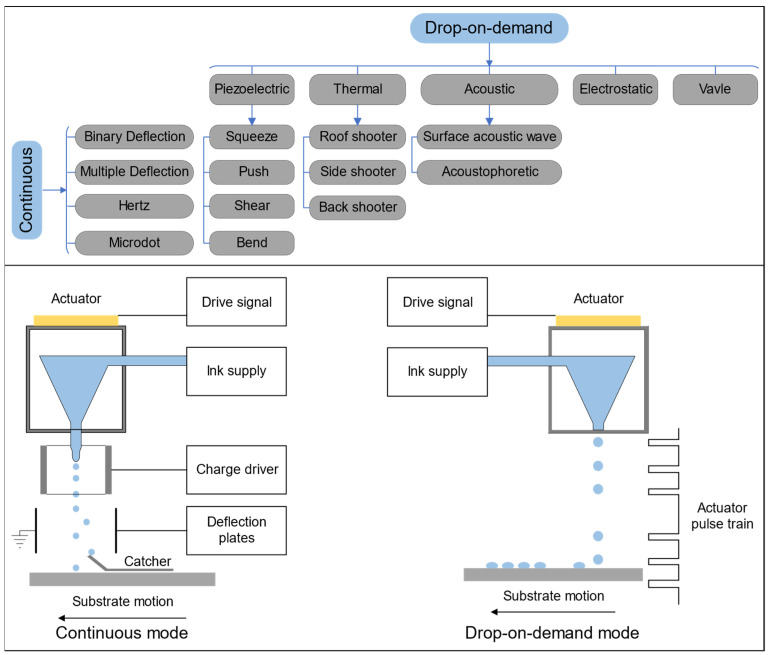
The classification of IJP [59].

**Figure 4:**
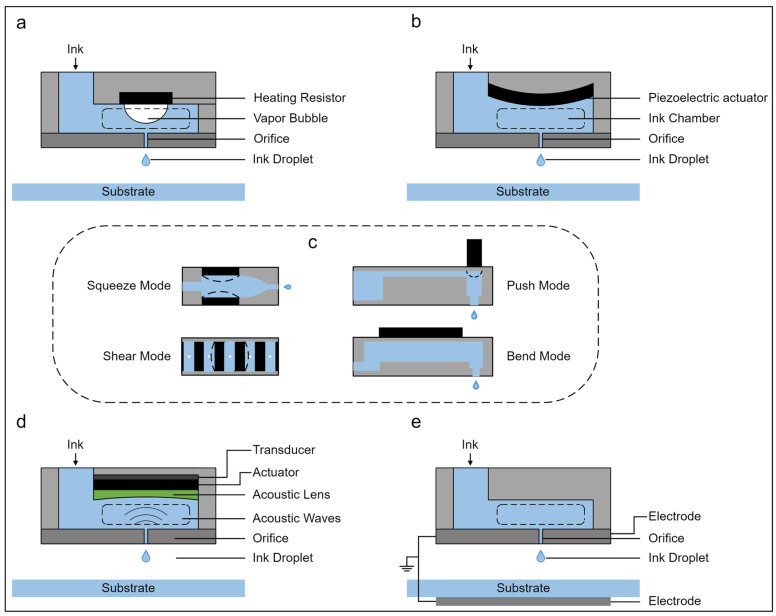
Schematics of IJP. (**a**) Thermal ink jetting. (**b**) PTZ ink jetting. (**c**) “Squeeze”, “push”, “shear”, and “bend” modes of PTZ ink jetting. (**d**) Acoustic ink jetting. (**e**) Electrostatic ink jetting [61,62,64].

The principles of IJP were first developed commercially during the 1970s and 1980s and applied practically to marking products with dates and codes and addressing mail [58]. The IJP process involves the ejection and local deposition of a sequence of droplets with fixed volume (typically from 10^−12^ L to 10^−9^ L) in either a continuous inkjet (CIJ) or drop-on-demand (DOD) inkjet mode (Figure 3) [59]. In CIJ printing, ink droplets are continuously ejected from the orifice and deflected afterward to write spots on the substrate, recycling the droplets that are not selected in this way via a gutter [60]. In DOD inkjet printing, ink droplets are ejected from thousands of orifices, typically arranged in each printhead as an array by inducing a transient pressure pulse within the chamber only when printing is needed [60]. A DOD inkjet printer can adopt several printheads with different driving mechanisms: piezoelectric (PTZ), thermal, acoustic, and electrostatic (Figure 4a,b,d,e) [61,62], among which piezoelectric and thermal printheads are most widely used. Alternatively, a thermal printhead relies on a resistor to heat the ink within the ink chamber until a bubble expands in it, forcing a droplet out of the orifice [63]. In the PTZ printhead, a PTZ actuator located near the orifice deforms under an applied voltage, leading to a sudden reduction in the ink chamber capacity, which creates a shockwave in the ink and ejects the droplet. The deformation of the PTZ element specifically determines the types of PTZ jetting, such as “squeeze”, “push”, “shear”, or “bend” (Figure 4c) [64].

**Figure 5:**
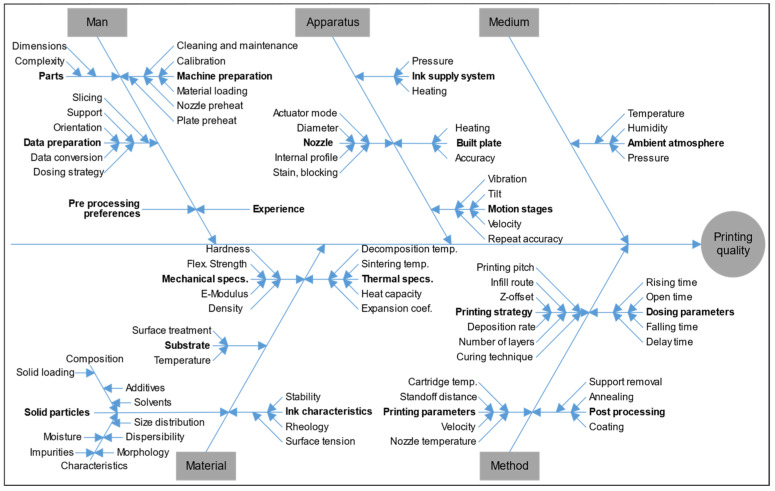
Various factors influencing the final quality of parts printed using MJ [65,66,67].

It is necessary to adequately understand the influence of various parameters on the quality of the final printed objects (Figure 5) [65,66,67] and establish optimal process conditions. A basic prerequisite of the IJP process reliability is the stability of functional inks and their specifications in terms of viscosity and surface tension, which negatively affect the shape and speed of ejected droplets as well as the spreadability on a substrate [54]. There are four major types of functional inks for IJP, namely phase change inks, solvent-based inks, water-based inks, and UV curable inks, whose viscosity is typically required to be less than 30 mPa·s to ensure fast refilling of the ink chamber (even under fast duty cycles) and droplet ejection from orifices [68]. Notably, some printers equipped with industrial PTZ printheads (i.e., Xaar1003, Xaar Nitrox, Konica KM1800i, and GALAXY series) and advanced recirculation systems allow functional inks with viscosities up to 80 mPa·s. The appropriate surface tension is in the range of 35–60 mN/m [69]. Unwanted dripping and ink spreading over the orifice plate that affects the jettability occur when surface tension is too low, whereas the jet stream may not split into individual droplets when surface tension is too high. Moreover, a balance of viscosity and surface tension should be guaranteed to prevent the formation of satellite droplets or ink mist. The dimensionless number Z = 1/Oh (Oh is the Ohnesorge number, calculated by Equation (3)) first defined by Fromm [70] is widely used to evaluate the printability of functional inks for IJP,

(1) $$We=\frac{v^{2}\rho d}{\gamma },\;$$

(2) $$Re=\frac{v\rho d}{\eta },$$

(3) $$oh=\frac{\sqrt{We}}{Re}=\frac{\eta }{\sqrt{\gamma \rho d}},$$

where ρ is the density of the ink, η is the viscosity of the ink, γ is the surface tension of the ink, d is the characteristic length (typically the diameter of the orifice), and v is the velocity of the droplet in flight. Reis et al. [71] proposed that droplets are stably ejected when 1 < Z < 10. At low values of Z, the viscous force will prevent droplet separation from the orifice, resulting in long thin tails and an extended period of time for single droplet ejection [72]. In turn, at high values of Z, the primary droplet would likely be accompanied by several satellite droplets. Derby [73] suggested this criterion can be used as guidance in the formulation of functional inks for IJP. Jang et al. [74] explored the piezoelectric inkjet process with various fluid mixtures of ethanol, water, and ethylene glycol by monitoring droplet formation dynamics and determined the reasonable range was 4 < Z < 14. However, because the above boundaries are semi theoretical and can be affected, for instance, by the printhead design or the use of a heated printhead, the formulated ink must be optimized for the ejection tests [75].

The properties (e.g., size distribution, shape, and volume fraction) of the particles dispersed in the base fluid, as well as their agglomeration, contribute to printing unreliability. On the one hand, dispersed particles smaller than ≈1/50 of the orifice diameter (typically 10–100 µm) can largely reduce the occurrence of clogging [76]. On the other hand, the size of dispersed particles is also strongly associated with the sedimentation velocity, as shown in Stokes law (Equation (4)) derived considering the movement of spherical particles in a base fluid,

(4) $$V_{s}=\frac{\left(\rho_{p}-\rho_{f}\right)gd^{2}}{18\eta_{0}}{\left(1-\phi \right)}^{n},$$

where $V_{s}$ is the sedimentation velocity, $\rho_{p}$ and $\rho_{f}$ are the densities of the particle and base fluid, respectively, g is the gravitational constant, d is the particle diameter, $\eta_{0}$ is the base fluid viscosity, ϕ is the solid (particle) volume fraction, and n is 4.75 when the particle diameter is greater than 2 µm and 5.25 for submicron [77,78]. Reducing the size of dispersed particles can reduce their sedimentation velocity and enhance the characteristics of the final printed targets in terms of density, strength, and dielectric properties after sintering [79,80,81,82,83,84]. However, the contact area and surface interaction between these dispersed particles would increase concomitantly (under the condition of the same solid volume fraction), resulting in high suspension viscosity; as a result, the particle size distribution is important. In addition, the solid volume fraction is within 20 vol.% in most cases [54,85]. There are some exceptions; for example, Wätjen et al. [86] reported an alumina ink with a solid volume fraction of 31.1 vol.%. It is desirable to obtain a functional ink with the highest possible solid loading (ϕ) and lowest possible viscosity (η) for printing uniform, dense, low shrinkage, and defect-free bodies. Various models have been proposed to determine the ϕ−η relationship [87,88,89,90,91,92,93,94,95]. Among them, the Krieger–Dougherty model (Equation (5)) is the most extensively used,

(5) $$\eta =\eta_{0}{\left(1-\frac{\phi }{\phi_{m}}\right)}^{-B\phi_{m}},$$

where η is the viscosity of the ink, $\eta_{0}$ is the viscosity of the vehicle, ϕ is the solid volume fraction, $\phi_{m}$ is the maximum solid volume fraction, and B is the “Einstein coefficient” or “intrinsic viscosity” with a value of 2.5. Therefore, the size distribution and solid volume fraction of dispersed particles should be considered on the basis of the above factors.

#### 2.1.2. Aerosol Jet Printing (AJP)

**Figure 6:**
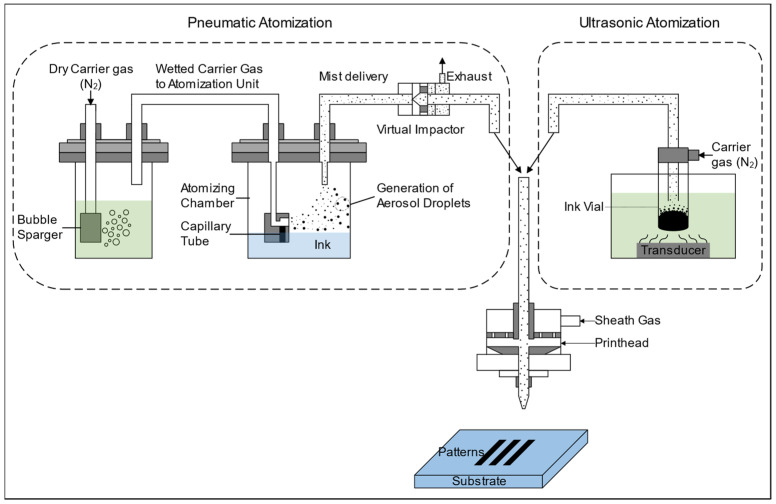
Schematics of AJP with both a pneumatic atomizer and ultrasonic atomizer.

Aerosol jet printing (AJP), formerly referred to as maskless mesoscale material deposition (M^3^D), was first developed under the Defense Advanced Research Projects Agency (DARPA) Mesoscopic Integrated Conformal Electronics (MICE) program to identify a manufacturing process capable of depositing a wide range of materials on any substrate [96]. Five core stages (atomization, aerosol transport, collimation, focusing, and impaction) are involved when an AJP system is working. Initially, fine mists of numerous aerosol droplets are generated by atomizers with pneumatic or ultrasonic approaches. Afterward, the carrier gas flow applied to the ink reservoir drives the aerosol droplets through a virtual impactor, where the aerosol droplets are filtered for more uniform size distribution and ultimately directed to the printhead. At the printhead, the coaxial sheath gas flow plays an important role, which collimates the aerosol droplets into a tight beam and forms an interlayer between the aerosol stream and physical components to reduce nozzle clogging. Finally, a high-velocity stream of collimated aerosol droplets, together with the sheath gas, exits from the printhead and is directed towards the substrate with a standoff distance of 1–5 mm (Figure 6).

Employing an ultrasonic transducer immersed in the water bath, the ultrasonic approach produces droplets smaller than 100 nm with a well-defined size distribution from small volumes (~2 mL) of relatively low viscosity (1–500 cp) inks [97] and diffuses these droplets into the gas phase. However, the underlying mechanism of liquid disintegration is not yet fully understood. There are two major hypotheses proposed to explain this phenomenon: cavitation and capillary wave [98]. The cavitation hypothesis considers that the cavitation activity, referred to as the nucleation, growth, and subsequent explosion of numerous microbubbles in the liquid layer caused by ultrasonic energy contributes to droplet generation [99]. In contrast, the capillary wave hypothesis based on the Taylor instability criteria states that droplet generation occurs when unstable oscillations (formed by high-frequency ultrasound) on the surface of the liquid column tear the crests of capillary waves away from the liquid layer [100]. In the pneumatic approach, during atomization, a compressed gas (carrier gas) flows across the top of an ink supply channel, creating a region of negative pressure that draws the bulk ink upward, where the bulk ink is impacted and sheared by the high-velocity carrier gas to generate aerosol droplets. The pneumatic approach is able to atomize inks with viscosities in the range of 1–1000 cp [101].

Human, material, apparatus, method, and medium factors affecting the AJP process are shown in Figure 5. As with IJP, the formulation of suitable ink is essential and is arguably the biggest challenge. However, a summary equation of parameters similar to the “Z number” has not been established, and initial formulation and optimization are achieved through large, isolated bodies of empirical work [97]. One of the requirements for ink formulation is that the solid particles do not clog the atomizer nozzle, which is usually achieved using submicron solid particles capable of deformation whose fragments or fines that become amorphized upon impact may facilitate consolidation [102]. Because the atomization process selectively introduces smaller particles into the aerosol stream, controlling a narrow particle size distribution is essential; otherwise, the particles in the ink available for printing will change as a function of printing time [103]. To prevent aerosol droplets from drying completely before delivery to the substrate, the solvent vapor pressure must be tuned; in many cases, the addition of a low volatility cosolvent (~10 vol.%) is necessary and empirical studies highlight its use in suppressing overspray [104].

Adjustable process parameters associated with the final quality of printed targets, such as sheath gas flow rate (SHGFR), carrier gas flow rate (CGFR), focusing ratio (FR, the ratio of sheath gas flow rate to carrier gas flow rate), standoff distance, stage speed, and substrate temperature have been extensively investigated [105,106,107,108,109]. Both the morphology and electrical performance of printed lines can be greatly affected by the poor optimization of these process parameters. Akhatov et al. [110] conducted the first systematic theoretical and experimental study about the effect of the Saffman force on aerosol flows through a micro-capillary, revealing complicated interactions between droplets and the carrier gas, which allowed for the optimization of focused aerosol beams. Later, many researchers explored the underlying causal aerodynamic interactions that led to trends in line morphology using computational fluid dynamic models and achieved a better understanding of aerosol droplet generation, transportation, and impaction [104,111,112,113,114,115,116]. Additionally, data-driven process modeling can eliminate the drifting and stochastic nature of the print system and efficiently determine the optimal operating process window, which is attractive for controlling the line morphology [117,118,119].

### 2.2. Direct Ink Writing (DIW)

**Figure 7:**
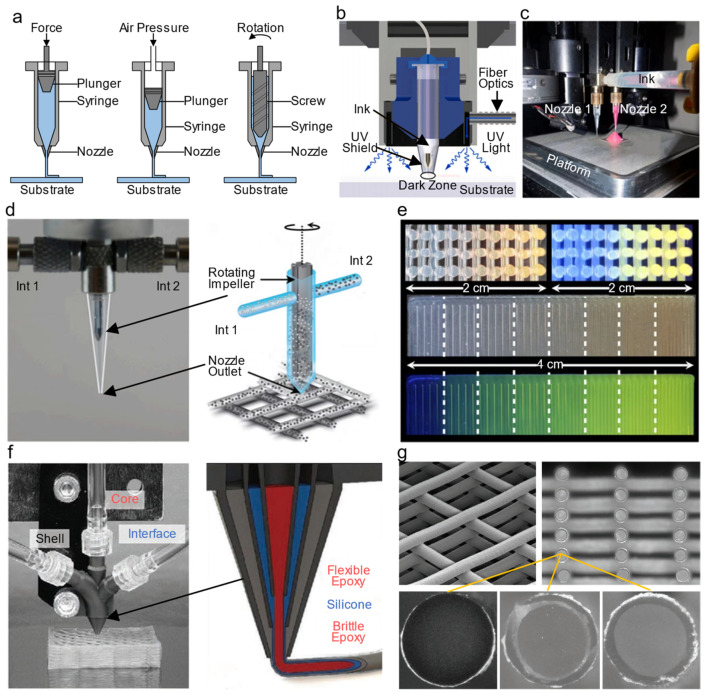
(**a**) Schematics of plunger-based extrusion, pneumatic-based extrusion, and screw-based extrusion of DIW. (**b**) Cross sectional view of the UV-assisted DIW process [128]. (**c**) Multi-material 3D printing with a dual nozzle DIW system [133]. (**d**) Optical image and schematic illustration of the impeller-based active mixer for multi-material DIW, and (**e**) cross sectional images of the printed 3D rectangular lattice structure showing continuous change in the fluorescent pigment concentration [134]. (**f**) Optical image and schematic illustration of the multicore-shell nozzle connected to the core, interface, and shell ink syringes for multi-material DIW, and (**g**) cross sectional images of the printed rectangular lattice structure [138].

DIW originated from robocasting technology, which was initially established by Cesarani et al. at Sandia National Laboratories in 1997. As an extrusion-based versatile 3D printing method, DIW provides a powerful route for fabricating complex 3D structures with high aspect ratio walls or spanning elements at the meso- and microscale from polymers (conductive or insulating) [120,121], ceramic particles [22,122], metal particles [123], graphene nanosheets [124], carbon nanotubes (CNTs) [125], and composites [126]. During printing, the ink (also called a suspension or paste) with shear thinning behavior is pneumatically or screw extruded through a motion-controlled nozzle (typical diameter 100–500 µm, Figure 7a) and then deposited on the platform to form precise patterns. To steadily stack multiple layers and guarantee fidelity and structural integrity, the extruded ink must fully solidify instantaneously, which is assisted by an optional post-curing process (solvent evaporation, gelation, solvent-driven reactions, thermal treatment, and photoirradiation) to allow for crosslinking [127]. Figure 7b shows a modified UV-assisted DIW setup [128].

Various material and process parameters, for instance, the rheological properties of the ink (e.g., viscosity and surface tension), the geometric features of the nozzle, extrusion pressure, standoff distance, printing speed, strip spacing, and curing technique, significantly dictate the quality (e.g., dimensional error, surface roughness, layer flatness, and interfacial strength) of the printed 3D parts [129,130]. Several essential criteria of qualified inks for DIW include the ability to flow easily through the nozzle when subjected to a shear force, sufficient yield strength after extrusion, and self-supportability. The feature resolution is positively correlated with the size of the nozzle. Although smaller nozzles provide higher feature resolution, they sacrifice build efficiency, and a greater extrusion pressure is required to enable smooth flow of the viscoelastic ink at the risk of nozzle damage. Notably, DIW with a nozzle diameter ranging from 0.5 µm to 5 µm has been used to create structures with feature sizes ranging from 100 nm to 10 µm [131].

There are three different strategies enabling multi-material 3D printing with DIW (multi-material DIW) according to the nozzle design and ink extrusion mode. The first is that multiple inks stored separately in their individual syringes are extruded through multiple nozzles to construct multi-material structures layer-by-layer (Figure 7c) [132,133]. This strategy is the most commonly used and involves switching between multiple nozzles for sequential printing, which requires accurate coordination of each nozzle and careful flow control of viscoelastic inks, especially when the printed inks have different rheological properties [21]. The second uses a single nozzle with the capability of switching between inks or in situ mixing of multiple inks via a mixing impeller driven by rotary motor during the printing process while simultaneously controlling the printed geometry (Figure 7d–e) [134,135,136]. This strategy makes it possible to fabricate functionally graded patterns with tunable properties (e.g., strength, coefficient of thermal expansion, and permittivity). The third strategy uses a multicore-shell nozzle with several specific inputs connected to corresponding syringes, which enables the coextrusion of multiple inks to form a core-shell filament (Figure 7f–g) [137,138].

Recently, multi-material DIW has been increasingly adopted to manufacture functionally graded structures (e.g., triboelectric nanogenerators [139], sensors [140], supercapacitors [141,142], photodetectors [143], batteries [132], circuit board [144], and scaffolds [145]) because of its unique ability to separately or simultaneously extrude different functional inks. For example, Sears et al. [145] used a multi-material DIW printer to fabricate a scaffold with a poly(ε-caprolactone) (PCL) or poly(lactic acid) (PLA) shell and a propylene fumarate dimethacrylate (PFDMA) hierarchical architecture. The addition of the PCL or PLA shell resulted in a significant increase in the compressive modulus and yield strength. Natural bone tissue comprises hierarchical porous structures that support cell growth, provide space for nutrient transport, and withstand different types of ambient loads [146]. Critical size or non-union bone defects can be treated by surgically implanting biocompatible bone graft substitutes [147]. However, there are many problems in the long-term use of biocompatible bone graft substitutes, such as displacement, allergic reaction, and the need for secondary surgery [148]. Another permanent solution is to facilitate bone tissue regeneration via a scaffold. Multi-material DIW has garnered considerable attention because of its potential in constructing the heterogeneous and anisotropic structure that can enable scaffolds to mimic the properties of natural bone tissue [123,149]. In addition, triply periodic minimal surface (TPMS) scaffolds allow for precise geometric modification; as a result, many physical characteristics (e.g., surface-to-volume ratio, pore size, elastic properties, and fluid behaviors) can be controlled [150].

### 2.3. Fused Deposition Modeling (FDM)

FDM is one of the most extensively used 3D printing techniques for polymer structures (e.g., polylactic acid, butadiene styrene, polycarbonate, polyamide, polystyrene, and polyethylene [151]) because of its inexpensive apparatus cost and easy implementation. FDM is also known as fused filament fabrication (FFF); in this process, the thermoplastic polymer filament is melted or softened in a liquefier head, and then the polymer is selectively extruded by the action of two counter rotating elements on the platform to build the 3D objects in a layer-on-layer manner. Over the past few years, thermoplastic polymer filaments reinforced by a variety of fillers, such as metals [152,153], ceramics [154,155,156,157], fibers [158,159], and bioactive glass [160], have been developed. With enhanced properties (e.g., mechanical strength, thermal conductivity, permittivity, dielectric loss, and biocompatibility) of these composite filaments, FDM greatly expands its application scope into electronic, automotive, aerospace, biomedical, and sports industries.

The critical parameters of FDM are filament-specific (e.g., thermal, mechanical, and rheological properties and diameter), operation-specific (e.g., temperature, speed, and structure of 3D-printed object), and apparatus-specific (e.g., number of extrusion heads, nozzle diameter, and gear force) [161,162]. Multi-material 3D printing with FDM (multi-material FDM) can be easily implemented using multiple extrusion nozzles, and the method has drawn increasing interest. However, some inherent disadvantages still exist when multi-material FDM is used to construct complex structures, such as low resolution accompanied by poor surface finish, insufficient bonding between adjacent sections, and slow build velocity [21]. Additionally, thermoplastic polymer filaments with high solid content are necessary for the fabrication of metal and ceramic parts because of the low shrinkage after sintering; However, the dramatic increase in stiffness and brittleness adversely affects the production and printing process [163]. Therefore, significant efforts should be devoted to overcoming these challenges.

### 2.4. Vat Photopolymerization (VP)

In the vat photopolymerization (VP) process, a liquid-state photopolymer material housed in a vat is selectively cured and patterned by light-initiated polymerization [10]. This class of technologies includes stereolithography (SLA), two-photon lithography (2PL) or two-photon polymerization (2PP), digital light processing (DLP), and continuous liquid interface production (CLIP).

As one of the oldest VP technologies established by Hull in 1984, SLA uses a UV laser beam scanning quickly along a controlled path to cure the irradiated photopolymer material within a vat from point to point. After one slice is printed, the build platform moves downward or lifts (depending on if the machine is a top-down or bottom-up structure) with a constant amount equaling exactly one-slice thickness in the z-direction for the next slice printing until the 3D part is finished [164]. Another important laser scanning technology, 2PL, differs from conventional (single-photon) SLA by exploiting the two-photon absorption (2PA) process to induce polymerization of the photopolymer material within the focal region of an ultrafast (femtosecond or picosecond) pulsed laser. Therefore, 2PL can be used to fabricate 3D microstructures with feature resolutions beyond the diffraction limit and free from the staircase effect caused by fluctuations of photopolymer materials [165]. However, 2PL shares the same common time-consuming issue as SLA and requires careful design of the highly transparent feedstock, especially when involving light-scattering additives such as ceramic particles [166].

In contrast to the aforementioned laser scanning technologies, DLP employs a digital light projector rather than a laser as the light source; as a result, a UV image corresponding to each layer shape can be projected over the surface of the photopolymer material within a vat. DLP considerably improves building efficiency because any design can be printed layer-by-layer instead of individually addressing one voxel. Projection micro stereolithography (PµSL) is a development from DLP using a liquid crystal display, a digital micromirror device, or liquid crystals on the silicon device as a dynamic mask and featuring superior resolution (up to 0.6 µm) [167]. CLIP is another development from DLP enabling continuous growth of a 3D part using an oxygen-permeable and UV-transparent window at the bottom of the photopolymer material vat [168]. Oxygen can travel through the window and mix into the liquid photopolymer material, forming a persistent liquid interface (oxygen dead zone) capable of inhibiting free radical photopolymerization. As a result, the build platform no longer needs to move up and down for each layer [169].

**Figure 8:**
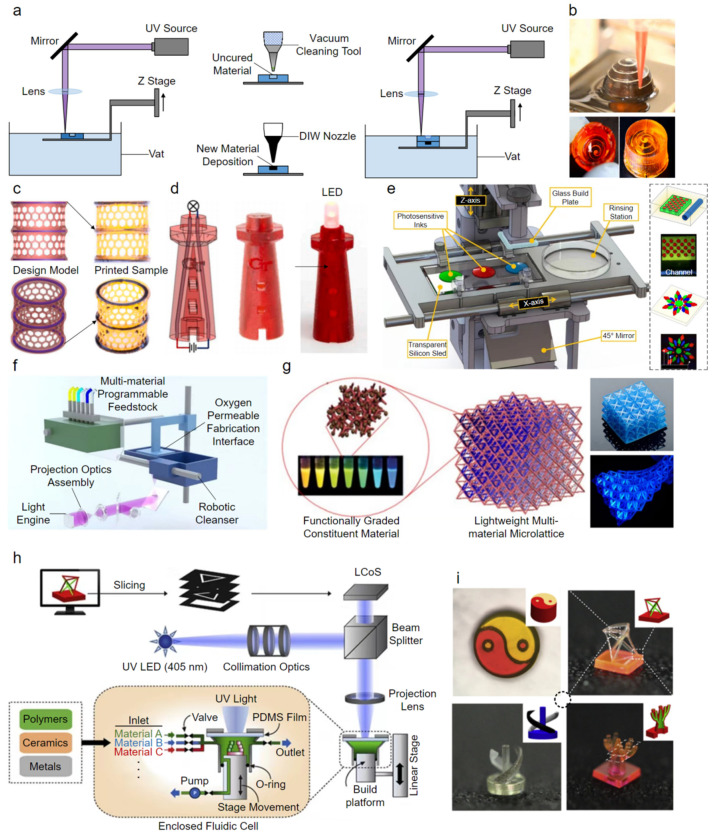
Multi-material 3D printing with VP-based technologies. (**a**) Schematic illustration of the multi-material 3D printing system integrating SLA and DIW. (**b**) Photographs of the printed electronic structure with an embedded 3D helix, (**c**) photographs of the printed cylindrical lattice with three rings embedded, and (**d**) photographs of the printed light tower [172]. (**e**) Schematic illustration of the multi-material stereolithography without vats [179]. (**f**) Schematic illustration of the multi-material stereolithography using an in situ microfuidic system, and (**g**) photographs of the printed 3D microlattice with dissimilar constituent materials [181]. (**h**) Schematic illustration of the multi-material PµSL system using dynamic fluidic control to rapidly fill and exchange multiple photopolymer materials, and (**i**) photographs of the printed micro 3D structures [183].

These VP-based 3D printing technologies have shown great potential in sensors, actuators, robots, microfluidic devices, and scaffolds because of their ability to construct 3D parts with high resolution, high accuracy, high throughput, and a good surface finish, which is not the case for MJ, DIW, and FDM [170]. However, most studies simply focus on single-material fabrication, and multi-material 3D printing with VP-based technologies remains challenging and limited because of difficulties in exchanging liquid-state photopolymer materials within a vat. In general, three different strategies have been developed: (1) a hybrid process via the combination with DIW, AJP, or other special techniques, (2) vat switching, and (3) dynamic fluidic control. Among a few recently reported applications, the first strategy may be the most straightforward and widely adopted. Lopes et al. [171] presented a hybrid multi-material 3D printing system that integrated SLA and DIW to fabricate monolithic structures with embedded circuits. SLA was used to build substrate structures, reserving the required receptacles into which electronic components were subsequently inserted, and channels and vertical vias were created, while DIW provided a precisely dispensing conductive material in the channels and vertical vias to realize interconnection (Figure 8a). Similarly, Peng et al. [172] presented a hybrid multi-material 3D printing system that integrated DLP and DIW to fabricate active soft robots, circuit-embedding architectures, and strain sensors (Figure 8b–d). This strategy requires an effective cleaning tool (e.g., air jet, vacuum, brush, and ultrasonic) to remove the uncured photopolymer material from reserved receptacles, channels, and vertical vias, as any material contamination leads to dimensional inaccuracies. With the second strategy, the simplest approach is to manually or automatically exchange the photopolymer material within a vat [173] or switch between multiple vats containing different photopolymer materials [174,175]; however, the printing process has to be interrupted, significantly increasing its time consumption. To overcome this issue, researchers have proposed multi-material stereolithography without vats that can deliver multiple photopolymer materials on a moving glass plate or silicone sled using aerosol jet systems [176] or pump-based syringes [177,178,179] (Figure 8e). In this distinctive process, a bottom-up projection method is mostly adopted, which greatly reduces the amount of residue to be cleaned because the printed part is immersed in the liquid-state photopolymer material at a limited depth [180]. Notably, in combination with an in situ microfuidic system (Figure 8f), Chen et al. [181] achieved 3D microlattices with dissimilar constituent materials (Figure 8g), which is attractive for manufacturing various structures with tailorable properties such as a negative Poisson’s ratio. Miri et al. [182] demonstrated an in situ microfuidic system with several on/off pneumatic valves for fast switching between different photopolymer materials and accomplished the multi-material stereolithography of heterogeneous hydrogel constructs. With the third strategy, recently, Han et al. [183] developed a multi-material PµSL system using dynamic fluidic control to realize rapid filling and exchange of multiple photopolymer materials within an integrated pressure-tight fluidic cell (Figure 8h) that is capable of fabricating highly complex heterogeneous 3D microstructures (Figure 8i).

## 3. Applications of Multi-Material 3D Printing in Functional Ceramic Devices

In most cases, functional ceramic devices with multiple material constituents have tiny structural features such as strip lines, microstrip lines, and vias. These structural features present a challenge for multi-material 3D printing processes because micro-defects will degrade the electrical and mechanical properties. In addition, the materials (ceramics, polymers, and metals) and their compatibility with 3D printing techniques must be further investigated. Table 2 lists several composition examples of multi-material 3D printing in functional ceramic devices.

### 3.1. Capacitors

**Figure 9:**
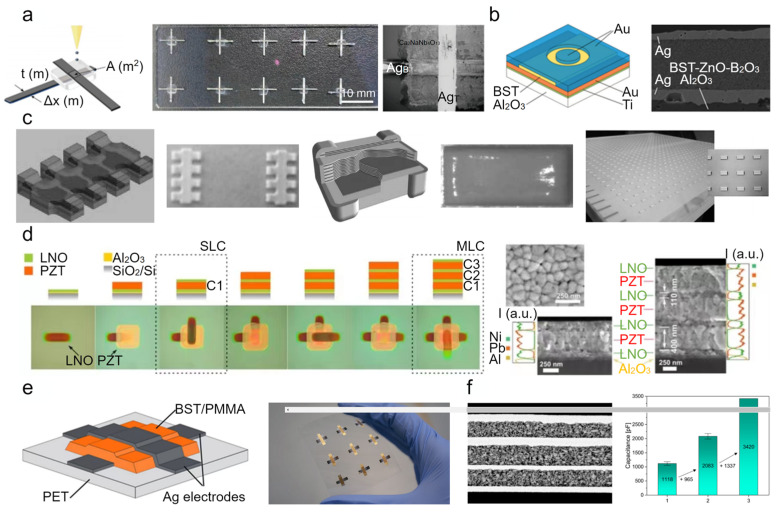
Multi-material 3D-printed ceramic capacitors. (**a**) Schematic illustration of the design concept of the Ag/Ca_2_NaNb_4_O_13_/Ag capacitor (**left**) and photograph of ten fully inkjet-printed Ag/Ca_2_NaNb_4_O_13_/Ag capacitors on a glass substrate (**middle**) and two overlapped Ag electrodes separated by the printed Ca_2_NaNb_4_O_13_ layer (**right**) [184]. (**b**) Schematic illustration of the design concept for the Ag/Ba_0.6_Sr_0.4_TiO_3_-ZnO-B_2_O_3_/Ag varactor (**left**), and SEM image of the fully inkjet-printed Ag/Ba_0.6_Sr_0.4_TiO_3_-ZnO-B_2_O_3_/Ag varactor (**right**) [185]. (**c**) Photographs of fabricated 784 MLCCs with 15 internal electrodes and, consequently, 14 dielectric layers using a multi-material 3D inkjet printer [186]. (**d**) Photograph of sequentially inkjet-printed LNO and PZT layers to form MLCC components (**left**), and SEM image of the fully inkjet-printed MLCC components on SiO_2_ substrate (**right**) [187]. (**e**) Schematic illustration of the design concept for the Ag/Ba_0.6_Sr_0.4_TiO_3_/PMMA/Ag capacitor (**left**) and a photograph of the fully inkjet-printed flexible capacitor array on PET substrate (**right**) [202]. (**f**) SEM image of the fully inkjet-printed MLCC component with three dielectric layers on a PET substrate (**left**) and increasing capacitances of the MLCC component with every additional layer (**right**) [205].

As the most widely used passive components in electronic equipment (i.e., smartphones, computers, and electric vehicles), ceramic capacitors can distribute and control the amount of current flowing through a circuit, eliminate noise, store energy, and prevent malfunction. Multi-material 3D printing enables ceramic capacitors to be fabricated directly from digital models, which provides an effective tool to enhance the flexibility of the substrate, material, and design, as well as reduce consumption and turnaround time [196]. Reviewing the recent research on multi-material 3D printing of ceramic capacitors, two major dielectric materials have been developed: pure ceramics [184,185,186,187,197,198,199] and ceramic/polymer composites [200,201,202,203,204,205,206,207]. For example, Rammal et al. [197] presented a fully inkjet-printed metal-insulator-metal (MIM) capacitor formed by layer-on-layer deposition of silver nanoparticle and ceramic nanoparticle (a low-temperature cofired ceramic material from Heraeus) based inks. The resulting 0.9 mm × 0.9 mm capacitor with a total thickness of 30 µm contained a 20 µm dielectric layer whose relative permittivity ε_r_ and dielectric loss tan δ were 67.5 ± 1 and 0.00128 ± 0.00006, respectively, leading to an approximate capacitance of 2 pF at 20 GHz. Fu et al. [184] fabricated a capacitor array comprising ten fully inkjet-printed MIM (Ag/Ca_2_NaNb_4_O_13_/Ag, where Ag terminal electrodes are on the top and bottom of the Ca_2_NaNb_4_O_13_ dielectric layer) capacitors on a glass substrate (Figure 9a), indicating that large area ceramic capacitors can be achieved at low cost using multi-material 3D printing. A capacitance density of ≈210 pF/mm^2^, with a low current density (<10^−7^ A cm^−2^) and low dielectric loss (≈0.02), was obtained for a capacitor with a ≈2 µm dielectric layer. Multi-material 3D printing has dramatically transformed the design and fabrication of tunable microwave devices such as varactors [185,208], phase shifters [209,210], and phased array antennas [211,212]. Friederich et al. [185] demonstrated a fully inkjet-printed MIM (Ag/Ba_0.6_Sr_0.4_TiO_3_-ZnO-B_2_O_3_/Ag) varactor, characterized by tunability between 14.4% and 16.4% under a tuning field of 5 V/µm (Figure 9b).

To achieve a higher capacitance density, parallel-plate capacitors are stacked to create multilayer components that have also been demonstrated via multi-material 3D printing. Jeschke et al. [198] proposed a fully inkjet-printed multilayer ceramic capacitor (MLCC) stacked by 5 Ag/barium titanate (BaTiO_3_)/Ag parallel-plate capacitors. The resulting MLCC component had a capacitance density of 1600 pF/mm^2^, nearly four times greater than that of a parallel plate capacitor. Dossou-Yovo et al. [186] investigated the methodology of MLCC component manufacturing by employing a CeraDrop multi-material 3D inkjet printer to alternately print Ag electrodes and MgTiO_3_ dielectric layers on a polymer substrate (to facilitate subsequent separation of the printed MLCC component from the platform), and obtained 784 MLCC components (each MLCC component incorporated 15 internal Ag electrodes and consequently 14 MgTiO_3_ dielectric layers) over a large area (Figure 9c). Folgar et al. [199] used the combination of AJP and selective laser sintering to synthesize MLCC structures (comprising 0.5–1.0 µm thick Ag electrodes and 0.5–2 µm thick BaTiO_3_ dielectric films) on a silicon substrate. Anti-diffusion layers of polymethyl methacrylate (PMMA) were used to prevent the nano silver ink from diffusing into the BaTiO_3_ layer. Matavž et al. [187] developed a highly efficient process to conformal stack ferroelectric oxide layers on silicon oxide substrates via inkjet printing. MLCC components comprising alternate layers of lanthanum nickelate (LNO) electrode and lanthanum-doped lead zirconate titanate (Pb_0.97_La_0.02_Zr_0.53_Ti_0.47_O_3_) dielectric film were demonstrated using this process (Figure 9d). For controlling the wetting behavior of functional inks on the previously formed layer, PMMA/polystyrene layers were used as temporary surface modification layers.

Over the past few decades, the demand for flexible electronics that require stretchable circuits to connect individual working units throughout the system has grown rapidly. Although these capacitors with pure ceramics as dielectric layers exhibit excellent permittivity and low dielectric loss, they are not suitable for flexible substrates because of their high annealing temperature (>850 °C) and poor bendability. Ceramic/polymer composites are promising candidate materials for capacitors embedded in stretchable circuits, and they have attracted attention because of their advantages of being synthesized from two original materials (high permittivity of the ceramics and mechanical properties and processability of the polymers). Mikolajek et al. [52] demonstrated 12 fully inkjet-printed MIM (Ag/Ba_0.6_Sr_0.4_TiO_3_/PMMA/Ag) capacitors on an alumina substrate. The Ba_0.6_Sr_0.4_TiO_3_/PMMA composite dielectric layer (about 7.2 µm) showed a relative permittivity ε_r_ of 28 ± 1.7 (approximately nine times greater than that of pure PMMA) and a dielectric loss tan δ of 0.043 ± 0.0006 at 10 kHz. The same authors subsequently demonstrated a fully inkjet-printed, flexible capacitor array on a polyethylene terephthalate (PET) substrate (Figure 9e) [202]. Lim et al. [203] presented a MIM capacitor on an alumina oxide/polymer composite substrate. The dielectric layer (with a relative permittivity ε_r_ = 70 and dielectric loss tan δ = 0.011 at 1 MHz) of this MIM capacitor was formed by resin infiltration into the micro-voids inside the inkjet-printed BaTiO_3_ film, and its thickness and roughness were 20 mm and 0.35 mm, respectively.

However, it is still a challenge to print very thin (<1 µm) and uniform dielectric layers that do not achieve the undesirable phenomenon known as the coffee stain effect during the drying stage [204]. To overcome this intrinsic problem, Reinheimer et al. [188] developed an innovative polymer/ceramic ink system comprising surface-modified (3-(trimethoxysilyl)propyl methacrylate) Ba_0.6_Sr_0.4_TiO_3_ particles, a cross-linking agent (poly(ethylene glycol) diacrylate), and a thermal radical initiator (dimethyl 2,2′-azobis(2-methylpropionate)), which allows immediate solidification of the ink. The fully inkjet-printed capacitor with a 700 nm dielectric layer showed a capacitance density of ≈500 pF/mm^2^. The same authors subsequently proposed a straightforward method to print MLCC components containing three dielectric layers on a PET substrate [205]. The obtained MLCC component, with three dielectric layers of 1.3 µm, 1.5 µm, and 1.2 µm, showed a capacitance up to 3420 pF, nearly three times greater than that of one parallel-plate capacitor (Figure 9f).

Various passive devices, such as capacitors, resistors, and inductors, are incorporated into chip packages or circuit boards to realize 3D system integration [213]. There have been significant efforts to build chip-first packages and interconnects using multi-material 3D printing [214,215,216,217,218]. For example, using a multi-material aerosol jet printer (Optomec Aerosol Jet 5X), Craton et al. [206] fabricated BaTiO_3_/polyimide composite films whose mixing ratios were dynamically adjusted during printing via an in situ mixing strategy, leading to tunable dielectric and mechanical properties (e.g., permittivity and coefficient of thermal expansion). Ring resonator circuits and MIM capacitors were printed on a molybdenum copper alloy (85% Mo and 15% Cu) carrier to characterize the BaTiO_3_/polyimide composite films. The same authors also demonstrated two chip-first microwave packages with integrated bypass capacitors on the same molybdenum copper alloy carrier [207], characterized by a maximum packaged gain of 21.7 dB and saturated output power of 21.9 dBm.

### 3.2. Multilayer Substrates

Multilayer ceramic substrates, also known as ceramic packages or shells, can satisfy the demand for 3D integration with active and passive components and high-reliability packaging. Multilayer ceramic substrates act as the medium for signal transmission and heat dissipation while also protecting chips from mechanical and chemical hazards, and they are widely used in the field of microelectronic packaging [219,220], sensors [221,222,223], electromagnetic (EM) absorbers [224,225], frequency selective surfaces (FSS) [226,227,228], and wideband near-field correcting [229].

**Figure 10:**
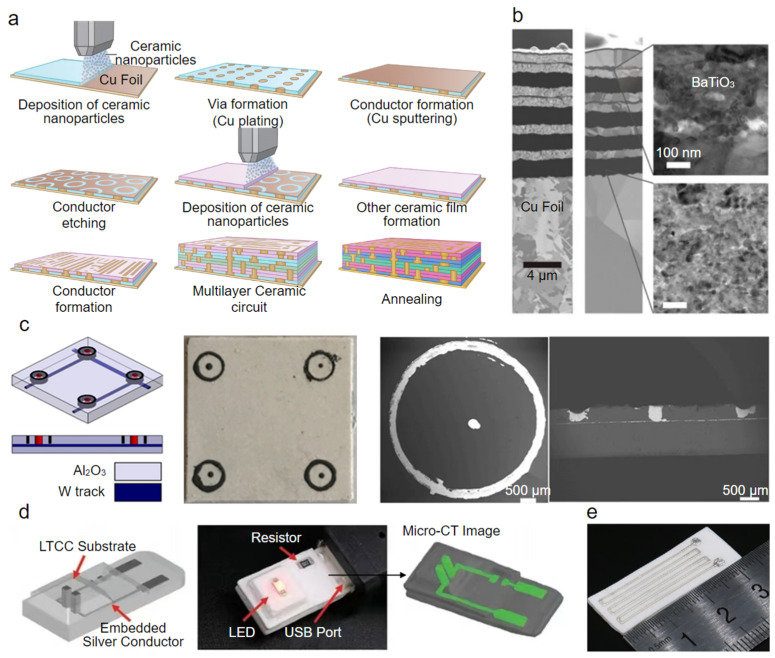
Multi-material 3D-printed multilayer ceramic substrates. (**a**) Schematic illustration of the hybrid multi-material integration process combining aerosol jet printing, chemical etching, sputtering, and plating techniques [190]. (**b**) SEM image of the fabricated mesoscale multilayer ceramic structure with fine copper electrodes and vias [190]. (**c**) Schematic illustration of the design concept for the HTCC structures (**left**) and photograph of the multi-material 3D-printed HTCC structure (middle) and its SEM image (**right**) [191]. (**d**) Schematic illustration of the design concept of the multilayer ceramic substrate for a LED (**left**) and photograph of the multi-material 3D-printed multilayer ceramic substrate in a working condition (**middle**) and its micro-CT image (**right**) [22]. (**e**) Photograph of a multi-material 3D-printed ceramic thermometer [22].

Multilayer ceramic substrates are mainly manufactured by HTCC or LTCC technology, which was originally derived from developments at RCA Corporation in the late 1950s [230]. To meet specific requirements, such as geometric complexity, microscale, multifunction, turnaround time, and cost-effectiveness, significant efforts have been devoted to realizing the multi-material 3D printing of multilayer ceramic substrates in the past few decades [22,189,190,191,231,232,233,234]. For example, Imanaka et al. [190] proposed a hybrid multi-material integration process using the combination of established AJP, chemical etching, sputtering, and plating techniques to fabricate the mesoscale multilayer ceramic structure with fine copper electrodes and vias (Figure 10a): employing AJP to deposit high-permittivity BaTiO_3_ films, chemical etching to selectively remove materials for shaping the via holes on the as-deposited BaTiO_3_ films and internal electrodes on the Cu sputter films, and plating to realize the metallization of via holes. Finally, to complete the multilayer ceramic structure (Figure 10b), a post-process annealing treatment was performed at 900–1000 °C in a nitrogen atmosphere. A permittivity of approximately 3000 and a dielectric loss of ≈7% at 1 MHz were demonstrated for the BaTiO_3_ film after annealing at 1000 °C. This multi-material integration process, with the help of multiple techniques, provides a direction for the manufacturing of a multilayer ceramic substrate.

Schulz et al. [232] developed alumina/glass composites with an optimized mixing ratio and particle size distribution and explored the multi-material 3D printing of LTCC substrates using a binder jetting printer. The internal conductors in X–Y and vertical directions were formed by depositing silver nanoparticles after the binder solidification of an aluminum oxide/glass dielectric layer. Despite the good permittivity (8.5) and dielectric loss (0.002) at 1 GHz, great improvements are needed because of the unsatisfactory shrinkage rate (X, Y ≈ 20%, Z ≈ 25%) and surface roughness (6.7–17.5) after cofiring. Compared with BJT, direct IJP is more suitable for fabricating multilayer ceramic substrates for higher resolution and better surface finish. Hirao et al. [189] formulated ceramic (BaO-Al_2_O_3_-SiO_2_-MnO-TiO_2_) and copper nanoparticle-based inks and deposited them together with a support material ink to construct multilayer ceramic structures, which were cofired at 800–1000 °C in a N_2_/H_2_ atmosphere, resulting in a shrinkage ratio of approximately 15%.

Multilayer ceramic structures have also been fabricated by coupling SLA with DIW. Raynaud et al. [233] developed a UV-curable ceramic slurry comprising alumina (d_50_ = 1.4 µm) and borosilicate glass (d_50_ = 1.6 µm) that was compatible with the SLA process and demonstrated the possibility of manufacturing LTCC structures via a multi-material 3D printer (from 3DCeram-Sinto company) integrating the SLA module and DIW module. The SLA module is used to form ceramic dielectric layers, where the channels and via holes are engraved by a pneumatic graver, while the DIW module is used to precisely deposit silver paste in these channels and via holes to form embedded metallic conductors in the X–Y and vertical directions. After cofiring for 1 h at 850 °C in an air atmosphere, the final dense multilayer ceramic structures showed a Young modulus of 83 ± 24 GPa and a flexural strength of 84 ± 24 MPa. The same authors subsequently proposed that HTCC structures (alumina) with tungsten electrodes can be manufactured through a similar multi-material 3D printing process (Figure 10c) [191]. Significant mismatching of the coefficient of thermal expansion (CET) between alumina (CET = 7.1 × 10^−6^/°C) dielectric and tungsten (CET = 4.10 × 10^−6^/°C) conductors would cause delamination at the ceramic/metal interfaces during co-firing. To reduce such delamination risk, Bernard et al. [234] replaced the tungsten paste used in experiments outlined by Raynaud et al. with molybdenum (CET = 5.35 × 10^−6^/°C) paste and demonstrated molybdenum conductors in the HTCC structures perfectly followed the profile of the alumina dielectric after cofiring. Indeed, such a solution coupling SLA with DIW is an advancement for multi-material 3D printing of multilayer ceramic structures (e.g., substrates, solid oxide fuel cells, and customized ceramic electronics); however, it requires an expensive apparatus. Recently, Wang et al. [22] demonstrated a simpler process employing multi-material DIW to fabricate multilayer ceramic structures for a LED substrate (Figure 10d) and thermometer (Figure 10e). During printing, two independent pneumatic dispensing devices alternatively extruded LTCC slurry and silver paste; once a ceramic layer with reserved trenches and hole vias was finished, the silver paste was filled in the trenches and hole vias to achieve coplanar circuitry and vertical interconnection.

### 3.3. Microstrip Antennas

**Figure 11:**
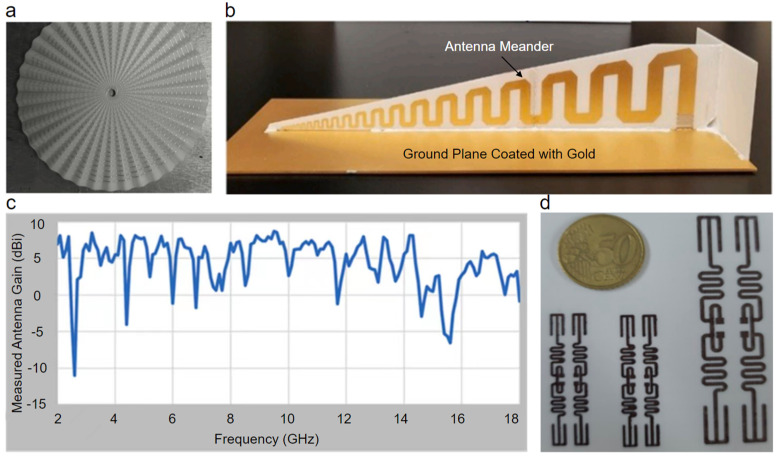
Multi-material 3D-printed ceramic microstrip antennas. (**a**) Photograph of the spiral microstrip antenna hybrid printed by NanoParticle Jetting™ and AJP [23]. (**b**) Photograph of the broadband microstrip antenna hybrid printed by DLP and AJP, and (**c**) measured gain versus frequency at room temperature [241]. (**d**) Photograph of the fully inkjet-printed antenna circuits [192].

Microwave (MW) dielectric ceramics are a research focus in the field of functional ceramics and have been widely used in numerous dielectric devices (i.e., filters [235], resonators [236], diplexers [237], power dividers [238], and antennas [239]) for modern wireless communication systems. The demand for simple, durable, cost-effective, low-profile ceramic microstrip (patch) antennas in the microwave frequency region has grown with recent advances in wireless communication [240]. Whether as standalone components or as components of arrays, multi-material 3D printing enables rapid prototyping manufacture of ceramic microstrip antennas with various sizes, complex structures, and material configurations. Many studies have been conducted to fabricate ceramic microstrip antennas through multi-material 3D printing. For example, Oh et al. [23] used the XJet Carmel 1400 printer to preliminarily print a zirconia dielectric substrate that was subsequently aerosol deposited with silver nanoparticle ink to form double spiral arms and finally produced a spiral microstrip antenna (Figure 11a). Similarly, Alhendi et al. [241] used DLP to preliminarily fabricate an alumina dielectric substrate that was subsequently aerosol deposited with gold nanoparticle ink to form metal patches and finally produced a broadband microstrip antenna (Figure 11b,c). The printed broadband microstrip antenna showed a gain of 8 dBi and a voltage standing wave ratio of 5 at 2 GHz. Although these two cases involving multi-material 3D printing of microstrip antennas are not a single-step process, they indicate great research potential. Microstrip antennas are compatible with printed-circuit technology; therefore, MJ techniques (IJP, AJP, and EHD) would be an excellent method to realize their single-layer or multilayer single-step manufacturing in a large area. Lee et al. [192] demonstrated antenna circuits through multi-material IJP (Figure 11d). The authors deposited photo-curable SiO_2_ nanoparticle ink to preliminarily fabricate a dielectric substrate and afterward deposited intense pulsed light sinterable Cu nanoparticle ink on it to form microstrip transmission lines.

Recently, the fabrication method and dielectric characteristics of the ceramic/polymer composite filaments for FDM of microwave devices (e.g., filters, antennas, and capacitors) have been widely investigated [154,155,156,157,193]. Castro et al. [194] formulated two types of ceramic/polymer composite filament using polydimethylsiloxane (PDMS, which is a common substrate material for stretchable electronic devices [242], such as transparent antennas [243], field-effect transistors [244], photodetectors [245], and energy harvesters [246], because of its thermal stability, high optical transparency, and capability of attaining designer functionalities via surface modification and bulk property tailoring) reinforced by NdTi0_3_ and MgCaTi0_2_ fillers with volume loading up to 25%. After determining the microwave dielectric properties of the two types of ceramic/polymer composite filament, the authors demonstrated flexible microstrip antennas via an nScrypt 3Dn Tabletop printer coupling FDM and DIW. The multi-material 3D-printed 19.6 GHz microstrip antenna showed a return loss of 20 dB along with a 10% bandwidth. The same authors subsequently formulated several types of high-permittivity and low-loss ceramic/polymer composite filament using cyclo-olefin polymer (COP) reinforced by MgCaTi0_2_, Ba_0.55_Sr_0.45_TiO_3_, and TiO_2_ fillers with different volume loading [195]. After determining the microwave dielectric properties of these different types of ceramic/polymer composite filament in detail, the authors demonstrated a multi-material 3D-printed Ku-band microstrip antenna with a dielectric substrate fabricated by FDM of COP-MgCaTiO_2_ (25 vol.%) composite filament. The antenna showed a peak realized gain of 6 dBi and patch area miniaturization of 50% compared with a reference antenna whose peak realized gain was 6.27 dBi. To enhance the microwave dielectric properties, the polymer is removed by sintering the ceramic/polymer composites or is reserved for a specific function (e.g., flexibility). The aforementioned examples of multi-material 3D printing of microstrip antennas would likely have additional applications in filters [247,248,249], resonators [234,250], and isolators [251].

## 4. Conclusions and Outlook

Multi-material 3D printing offers an excellent tool to directly fabricate 3D parts with material diversity that satisfy specific requirements, such as geometric complexity, microscale, multifunction, turnaround time, and cost-effectiveness, as well as make it possible to create gradient structures with tunable dielectric or mechanical properties. Despite advances in multi-material 3D printing over the past few years, there are still many challenges to be addressed. Among these multi-material 3D printing technologies, FDM is the easiest to implement with the lowest cost; however, poor surface finish and printing resolution can hardly expand its applicability in various fields in which structural conformability and dynamic configuration are required. In addition, materials need to be made into thermoplastic rod-like filaments, which greatly reduces the variety of materials that are available for printing. DIW has the strongest material adaptability, and it performs better in surface finish and printing resolution than FDM, particularly when using micro nozzles; however, the throughput is thus affected. MJ is more appropriate for fabricating miniature parts, and its non-contact property is beneficial to create submicro fine features; however, formulating functional inks with distinguished properties (e.g., rheology and electrical and mechanical characteristics) is important, and this remains a challenge with MJ. VP-based techniques offer the best solution that does not require support materials considering its outstanding surface finish, printing resolution, and throughput; however, it is generally not appropriate for multi-material 3D printing because of the difficulties of exchanging liquid-state photopolymer materials within a vat. In addition to technical breakthroughs considering the above factors, a deeper understanding of the fundamental materials and building process is needed to push the boundary of multi-material 3D printing.

Customized electronics have been a key driver for the development of multi-material 3D printing technology and may continue to be a commercial motivation. In the majority of cases using multi-material 3D printing technology to fabricate 3D complete circuitry, resin materials are more commonly used and perform better than ceramic materials. The key reason is that multi-material 3D-printed green bodies need to be debinded and cofired at different temperature stages when using ceramic materials. Significant mismatching of CET between xenogeneic materials (ceramics and metals) is likely to cause micro cracks and overall deformation, which affects the ultimate dimensional accuracy, flatness, and performance of ceramic devices. Therefore, xenogeneic materials should be compatible with multi-material 3D printing technologies and also establish their own property matching. Continuous efforts are needed to develop novel, advanced, and diverse raw materials to sustain the growth and adoption of multi-material 3D printing of functional ceramic devices. In addition, embedded vias, especially those with micron size, are themselves obstacles and challenges for multi-material 3D printing. It is necessary to exploit innovative and versatile multi-material 3D printing approaches with high resolution, high precision, and cost-effectiveness.

## Figures and Tables

**Table 1:** Summary of recently developed functional inks.

Types of Functional Inks	Printing Methods	Applications	Electrical Properties
Metal nanoparticles (MNPs) inks	Sliver MNPs inks; IJP [33], EHD [31]	Wearable electronics [31,33]	0.08–4.74 Ω sq^−1^ after 1 h of thermal sintering at 150 °C [33]; 0.4 Ω sq^−1^ after 30 min of thermal sintering at 250 °C [31]
	Gold MNPs inks; IJP [34], AJP [35]	Non-enzymatic electrochemical sensors [34]; micro-hotplates [35]	0.06 Ω cm^−1^ after 30 min thermal sintering at 100 °C [34]; 8.7 ± 2.5 μΩ cm after 1 h of thermal sintering at 120 °C followed by 250 °C for 4 h [35]
	Copper MNPs inks; IJP [36], EHD [37]	Conductive patterns and tracks [36]; micro-electronic devices [37]	6.18 Ω sq^−1^ after applying 5454 J energy [36]; 9.20 µΩ cm after 1 h of thermal sintering at 230 °C in inert atmosphere [37]
	Zinc MNPs inks; IJP [38], AJP [39]	Flexible electronics [38]; bioresorbable electronics [39]	~10^2^ S cm^−1^ [38]; 22.32 S cm^−1^ was achieved after 2 ms of sintering by 1 flash with energy of 25.88 J/cm^2^, and the final conductivity of 34.72 S cm^−1^ was achieved by an optimum laser power [39]
Conductive polymer inks	PEDOT: PSS inks; IJP [40], AJP [41]	Organic solar cells [40]; μ-needle electrode arrays [41]	0.02 S cm^−1^ after 20 min of thermal annealing at 120 °C (120 nm thick) [40]; 0.323 ± 0.075 S cm^−1^ [41]
	BBL: PEI inks; Spray-coating [42]	Organic electrochemical transistors and bioelectronics [42]	8 S cm^−1^ after 2 h of thermal annealing at 140 °C inside a nitrogen-filled glovebox [42]
Ceramic nanoparticle (CNP) inks	Al_2_O_3_ CNPs inks; IJP [43]	Thin film radio-frequency capacitors [43]	The dielectric constant of the printed alumina layer (~120 nm thick with ~0.5 nm RMS surface roughness after the thermal annealing at 400 °C) was 6.2 [43]
	BaTiO_3_ CNPs inks; IJP [44], AJP [45]	Piezoelectric generators [44]; interdigitated capacitors [45]	The piezoelectric generator had an open-circuit voltage of ~7 V, a current density of 0.21 μA·cm^−2^, and a power density of 0.42 μW·cm^−2^ [44]; the dielectric constant was 7 [45]
	3Y-TZP CNPs inks; IJP [46,47]	Dielectric films for microelectronic devices [46,47]	/
	ZrO_2_ CNPs inks; IJP [48], EHD [49]	Dielectric layers for flexible electronics [48]; resistive switches [49]	The ZrO_2_ dielectric film (dielectric constant of 10) afforded a leakage current density of 5.4 × 10^−6^ A/cm^2^ at 1 MV/cm [48]. The printed resistive switch showed stable bipolar memristive switching behavior around ± 3 V [49]
	TiO_2_ CNPs inks; IJP [50,51]	Mesoporous TiO_2_ electron transport layers for perovskite solar cells [50]; dielectric layers [51]	The perovskite solar cell had a power conversion efficiency of 18.29% [50]; the current-voltage characteristics of conducting oxide-TiO_2_-Ag devices showed diode behavior [51]
	Ba_0.6_Sr_0.4_TiO_3_ CNPs inks; IJP [52]	Dielectric layers for capacitors [52]	The relative dielectric constant was 28 ± 1.7, and the dielectric loss was 0.043 ± 0.006 (at 10 kHz) [52]
	Ca_2_Nb_3_O_10_ CNPs inks; AJP [53]	Thin-film transistors [53]	The films deposited by Ca_2_Nb_3_O_10_ ink with a mass fraction of 82 wt% showed a dielectric constant of 8.5 and a dielectric loss of 0.058 (at 1 MHz) [53]
	Glass silicate CNPs inks; IJP [54]	Multilayer hybrid circuits [54]	/
Dielectric polymer inks	Polyimide (PI) inks; IJP [55]	Capacitors for microelectronic devices [55]	The printed capacitor with 25 ± 0.2 µm thick PI layer showed a capacitance value of 103 pF [55]
	Poly 4-vinylphenol (PVP) inks; IJP [56]	Flexible capacitors for wearable electronics [56]	The printed capacitor with 4.5 µm thick PVP layer showed a capacitance value of 163 pF [56]
	Polyvinyl alcohol (PVA) inks; EHD [57]	Gate insulators in organic field-effect transistors [57]	The organic field-effect transistors with PVA-based gate insulators show stable operation with low gate leakage currents [57]

**Table 2:** Several composition examples of multi-material 3D printing in functional ceramic devices.

Compositions	Multi-Material 3D Printing Techniques	Applications	Properties	Ref.
Dielectric material (ink): Ca_2_NaNb_4_O_13_ + Isopropanol + 2-butyl alcohol Electrode material (ink): Ag	IJP	Capacitors	The capacitor showed a capacitance density of ≈210 pF/mm^2^	[184]
Dielectric material (ink): Ba_0.6_Sr_0.4_TiO_3_-ZnO-B_2_O_3_ + Butyl diglycol + isopropyl alcohol + ethyl cellulose Electrode material (ink): Ag	IJP	Varactors	The varactors showed a tunability between 14.4% and 16.4% under a tuning field of 5 V/µm	[185]
Dielectric material (ink): MgTiO_3_ Electrode material (ink): Ag	IJP	Capacitors	/	[186]
Dielectric material (ink): Pb_0.97_La_0.02_Zr_0.53_Ti_0.47_O_3_ + ethylene glycol + ethanolamine Electrode material (ink): Ag	IJP	Capacitors	/	[187]
Dielectric material (ink): Ba_0.6_Sr_0.4_TiO_3_ + Poly (ethylene glycol) diacrylate Electrode material (ink): Ag	IJP	Multilayer ceramic capacitors	The multilayer ceramic capacitors showed a capacitance density of ≈ 500 pF/mm^2^	[188]
Dielectric material (ink): BaO-Al_2_O_3_-SiO_2_-MnO-TiO_2_ Electrode material (ink): Cu	IJP	Multilayer ceramic substrates	The multilayer ceramic substrate showed a shrinkage ratio of ≈15%	[189]
Dielectric material (ink): BaTiO_3_ Electrode material (/): Cu	AJP + etching + sputtering + plating	Multilayer ceramic substrates	The multilayer ceramic substrate showed a permittivity of ≈ 3000 and a dielectric loss of ≈ 7% at 1 MHz	[190]
Dielectric material (slurry): Al_2_O_3_ Electrode material (slurry): tungsten	SLA + DIW	Multilayer ceramic substrates	The multilayer ceramic substrates showed a Young modulus E of ≈ 280 ± 11 GPa	[191]
Dielectric material (ink): ZrO_2_ Electrode material (ink): Ag	IJP + AJP	Microstrip antennas	The bulk ZrO_2_ showed a relative permittivity of 23 and a loss tangent of 0.0013 at microwave frequencies	[23]
Dielectric material (ink): SiO_2_ + hexanediol diacrylate (HDDA) + alkyl-diphenyl oxide disulfonate Electrode material (ink): Cu	IJP	Microstrip antennas	The resistance was 2.43 × 10^13^ Ω·cm (174.3 µm thick dielectric layer)	[192]
Dielectric material (filament): TiO_2_ + cyclo-olefin polymer (COP) Electrode material (slurry): Ag	FDM + DIW	Microstrip antennas	The 30% loaded COP-TiO_2_ showed a relative permittivity of 4.56 and a loss tangent of 0.0016 after sintering at 1100 °C	[193]
Dielectric material (filament): NdTi0_3_ + polydimethylsiloxane (PDMS) Electrode material (slurry): Ag	FDM + DIW	Microstrip antennas	The 25% loaded PDMS-NdTiO_3_ showed a permittivity of 9.22 and a loss tangent of 0.025 at frequencies up to 17 GHz	[194]
Dielectric material (filament): MgCaTi0_2_ + PDMS Electrode material (slurry): Ag	FDM + DIW	Microstrip antennas	The 19.6 GHz microstrip antenna showed a return loss of 20 dB along with a 10% bandwidth	[195]

## Data Availability

Not applicable.
